# Endoscope-Assisted Thoracic Duct Ligation and Cisterna Chyli Ablation Using Double O-Ring Wound Retractors in a Dog with Presumed Idiopathic Chylothorax

**DOI:** 10.3390/vetsci13090908

**Published:** 2026-09-04

**Authors:** Dongwoo Kim, Hyunsong Lee, Sang-Kwon Lee, Min Jang, Sung-Ho Yun, Young-Sam Kwon, Jinsu Kang

**Affiliations:** 1 Department of Veterinary Surgery, College of Veterinary Medicine, Kyungpook National University, 80, Daehak-ro, Daegu 41566, Republic of Korea; eastox6490@gmail.com (D.K.); hslee791@naver.com (H.L.); jangmin@knu.ac.kr (M.J.); shyun@knu.ac.kr (S.-H.Y.); kwon@knu.ac.kr (Y.-S.K.); 2 Department of Veterinary Medical Imaging, College of Veterinary Medicine, Kyungpook National University, 80, Daehak-ro, Daegu 41566, Republic of Korea; sklee10@knu.ac.kr

**Keywords:** chylothorax, O-ring wound retractor, endoscopic assistance, thoracic duct ligation, cisterna chyli ablation, dog

## Abstract

Chylothorax is a challenging condition in dogs, and although endoscopic thoracic duct ligation (TDL) combined with cisterna chyli ablation (CCA) is an established surgical treatment, adequate operative exposure and visualization remain technically demanding. This report describes the use of double O-ring wound retractors (OWRs) with endoscopic assistance to perform TDL and CCA in a dog with presumed idiopathic chylothorax. A 5-year-old castrated male French Bulldog presented with dyspnea caused by chylous pleural effusion. Computed tomographic lymphangiography identified abnormal thoracic duct branching with collateral lymphatic vessels and focal contrast leakage, supporting the diagnosis of presumed idiopathic chylothorax. Surgical treatment was performed through a right paracostal abdominal approach and a right circumcostal transdiaphragmatic approach using double OWR. Following methylene blue injection into a mesenteric lymph node, the thoracic duct and cisterna chyli were visualized endoscopically, and thoracic duct ligation, cisterna chyli ablation, and pleural port placement were performed under endoscopic visualization using conventional surgical instruments. The dog recovered uneventfully, with pleural effusion becoming radiographically undetectable by POD 34 and remaining absent on thoracic radiography at POD 48. No major postoperative complications were observed during follow-up. At POD 150, the owner reported no respiratory abnormalities or other clinical signs suggestive of recurrent chylothorax. This case demonstrates the technical feasibility of incorporating endoscopic assistance into a previously described double OWR-assisted approach for TDL and CCA in a dog with presumed idiopathic chylothorax. Because this technique was evaluated in a single dog without objective comparative assessment, the present report is intended to describe technical feasibility rather than demonstrate improved surgical or clinical outcomes.

## Simple Summary

Chylothorax is a condition in which lymphatic fluid accumulates within the chest cavity, causing breathing difficulty and reduced quality of life in dogs. Surgical treatment commonly involves thoracic duct ligation combined with cisterna chyli ablation, but these procedures can be technically challenging because of limited surgical exposure and difficulty identifying lymphatic structures. In this report, we describe the successful treatment of presumed idiopathic chylothorax in a French bulldog using an endoscope-assisted thoracic duct ligation and cisterna chyli ablation technique with double O-ring wound retractors. The wound retractors provided access to the surgical field and allowed visualization of the thoracic duct and cisterna chyli under endoscopic assistance while permitting the use of conventional surgical instruments. The dog recovered without major complications, and pleural effusion became radiographically undetectable by 34 days after surgery. At a telephone follow-up on postoperative day 150, the dog remained clinically stable, with no respiratory abnormalities or other clinical signs suggestive of recurrent chylothorax. This case demonstrates the technical feasibility of incorporating endoscopic assistance into a previously described double O-ring wound retractor-assisted approach for thoracic duct ligation and cisterna chyli ablation in a dog with presumed idiopathic chylothorax.

## 1. Introduction

Chylothorax is characterized by accumulation of chyle within the pleural cavity and is classified as either secondary to an underlying disease process or idiopathic [1]. In dogs with idiopathic chylothorax, thoracic duct ligation (TDL) is regarded as the standard surgical treatment, although reported success rates for TDL alone remain limited [2,3]. Cisterna chyli ablation (CCA) is commonly performed in combination with TDL to reduce lymphatic pressure and promote development of alternative lymphatic drainage pathways, which may improve surgical outcomes [4,5,6].

A single paracostal approach with transdiaphragmatic access for combined TDL and CCA was previously described in experimental and clinical dogs [7]. This approach was subsequently modified using double O-ring wound retractors (OWRs), which provided stable circumferential exposure and facilitated access to the thoracic duct and cisterna chyli [8]. In addition, various endoscopic and minimally invasive techniques have been described for TDL and CCA, including approaches performed individually or in combination [9,10,11]. The present case describes the use of endoscopic visualization through a double OWR-assisted paracostal and transdiaphragmatic approach, with the aim of facilitating identification and intraoperative assessment of the thoracic duct and cisterna chyli.

## 2. Case Presentation

A 5-year-old castrated male French bulldog weighing 13.4 kg was presented to a primary care veterinary hospital with tachypnea 10 days prior to referral. Thoracocentesis yielded approximately 1400 mL of pleural effusion, and chylothorax was diagnosed. The dog was subsequently referred to the authors’ institution because of persistent tachypnea and exercise intolerance. Following admission, thoracocentesis, echocardiography and computed tomography (CT) evaluation were performed, and conservative medical management including routine dietary modification was initiated.

On physical examination, the dog exhibited marked tachypnea (respiratory rate approximately 60 breaths/min). Serum biochemistry revealed mild elevation of alanine aminotransferase activity, with all other parameters within normal limits. Thoracocentesis yielded hemorrhagic chylous effusion. Biochemical analysis showed a pleural fluid triglyceride concentration of >500 mg/dL, compared with 76 mg/dL in serum, supporting the diagnosis of chylothorax. Pleural fluid and serum cholesterol concentrations were 79 and 183 mg/dL, respectively.

Echocardiography demonstrated a normal left atrial size (left atrial-to-aortic root ratio approximately 1.2) with mild mitral regurgitation, but no evidence of clinically relevant valvular degeneration, right atrial abnormality, or pulmonary arterial flow disturbance. A cardiogenic cause of chylothorax was therefore considered unlikely.

**Figure 1:**
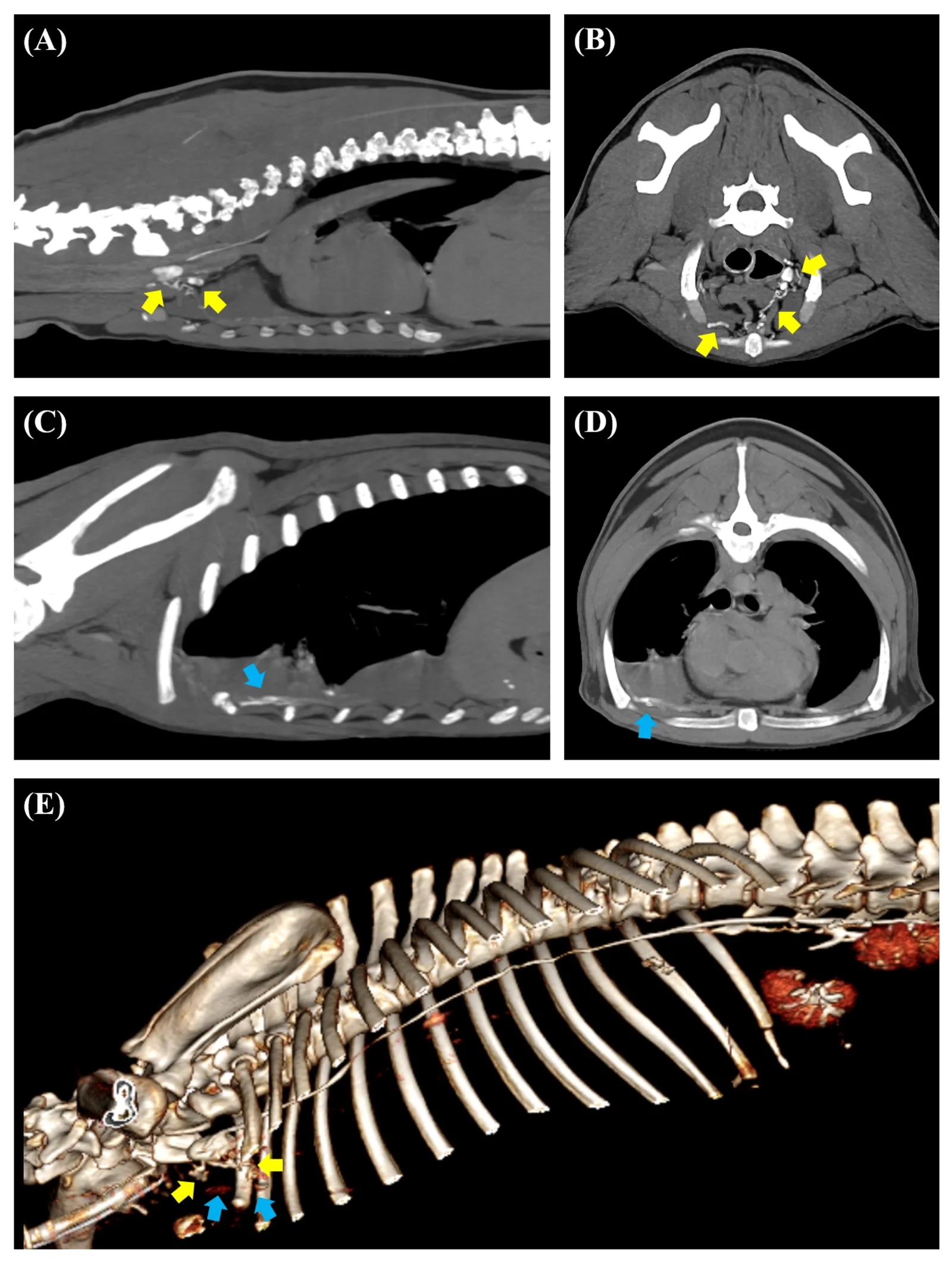
CT lymphangiography of the patient. (**A**,**B**) Multiple tortuous lymphatic branches (yellow arrows) arise from the thoracic duct ampulla and course cranially within the cranial mediastinum. (**C**,**D**) One of these branches extends along the ventral aspect of the right thoracic cavity, with multiple small branches and areas of suspected contrast leakage (blue arrows) surrounding the lymphatic vessels. (**E**) Three-dimensional volume-rendered reconstruction of the CT lymphangiographic image.

CT (Alexion16 TSX-034A, Toshiba, Kawasaki, Japan) identified bilateral pleural effusion with pulmonary atelectasis, without evidence of mediastinal mass or neoplasia. CT lymphangiography was performed by direct injection of iohexol (Omnipaque 300 Inj.; GE Healthcare, Norway; 300 mg I/mL) into the bilateral metatarsal pads at a dose of 4 mL per site. Following completion of contrast administration, both metatarsal pad injection sites were manually massaged to facilitate lymphatic uptake. CT acquisition was performed 4 min after completion of contrast injection, with an additional scan obtained 10 min after injection. CT images were acquired at 120 kVp and 120 mAs with a rotation time of 600 ms and a slice thickness of 1 mm, and images were reconstructed at a 1 mm slice thickness using a mediastinal reconstruction kernel (window width, 400 Hounsfield units [HU]; window level, 40 HU). Continuous contrast enhancement of the abdominal lymphatic vessels, cisterna chyli, and thoracic duct was identified. The thoracic duct coursed along the right dorsolateral aspect of the aorta before crossing to the left side and terminating at the thoracic duct ampulla. Multiple tortuous branches arose from the ampulla, including a branch extending along the ventral aspect of the right thoracic cavity with suspected contrast leakage (Figure 1). Mild pleural thickening and rounded atelectasis suggested chronic pleural inflammation.

Based on these findings, presumed idiopathic chylothorax was diagnosed, and surgical management with TDL and CCA was planned. A single paracostal approach using double OWR (VAXCON, Seoul, Republic of Korea) was selected to facilitate combined abdominal and thoracic access. Endoscopic assistance was planned to facilitate visualization of lymphatic structures during the procedure, particularly given the patient’s barrel-chested conformation.

Perioperative premedication consisted of fentanyl (3 µg/kg IV) and medetomidine (10 µg/kg IM). Anesthesia was induced with propofol (5 mg/kg IV) and maintained with sevoflurane in oxygen. Fluid therapy was administered using a balanced crystalloid solution (Plasma-Lyte; Plaju OP injection, JW Pharm., Republic of Korea) at a rate of 5 mL/kg/h. Fentanyl was administered as a constant rate infusion (CRI), with the rate adjusted according to intraoperative anesthetic and cardiovascular responses (range, 2–10 µg/kg/h). A transversus abdominis plane block was performed using 0.5% bupivacaine (0.2 mL/kg, diluted 1:1 with saline). Physiological variables were monitored using a multiparameter monitor (CARESCAPE Monitor B650; GE Healthcare, Helsinki, Finland), including heart rate, electrocardiography, pulse oximetry, esophageal temperature, and systolic, diastolic, and mean arterial blood pressures (SAP, DAP, and MAP, respectively). For invasive arterial blood pressure monitoring, an over-the-needle catheter was placed in the right dorsal pedal artery. End-tidal carbon dioxide (EtCO_2_), respiratory rate (RR), and the expired fraction of sevoflurane (FE′Sevo) were measured using a side-stream infrared gas analyzer incorporated into the multiparameter monitor.

**Figure 2:**
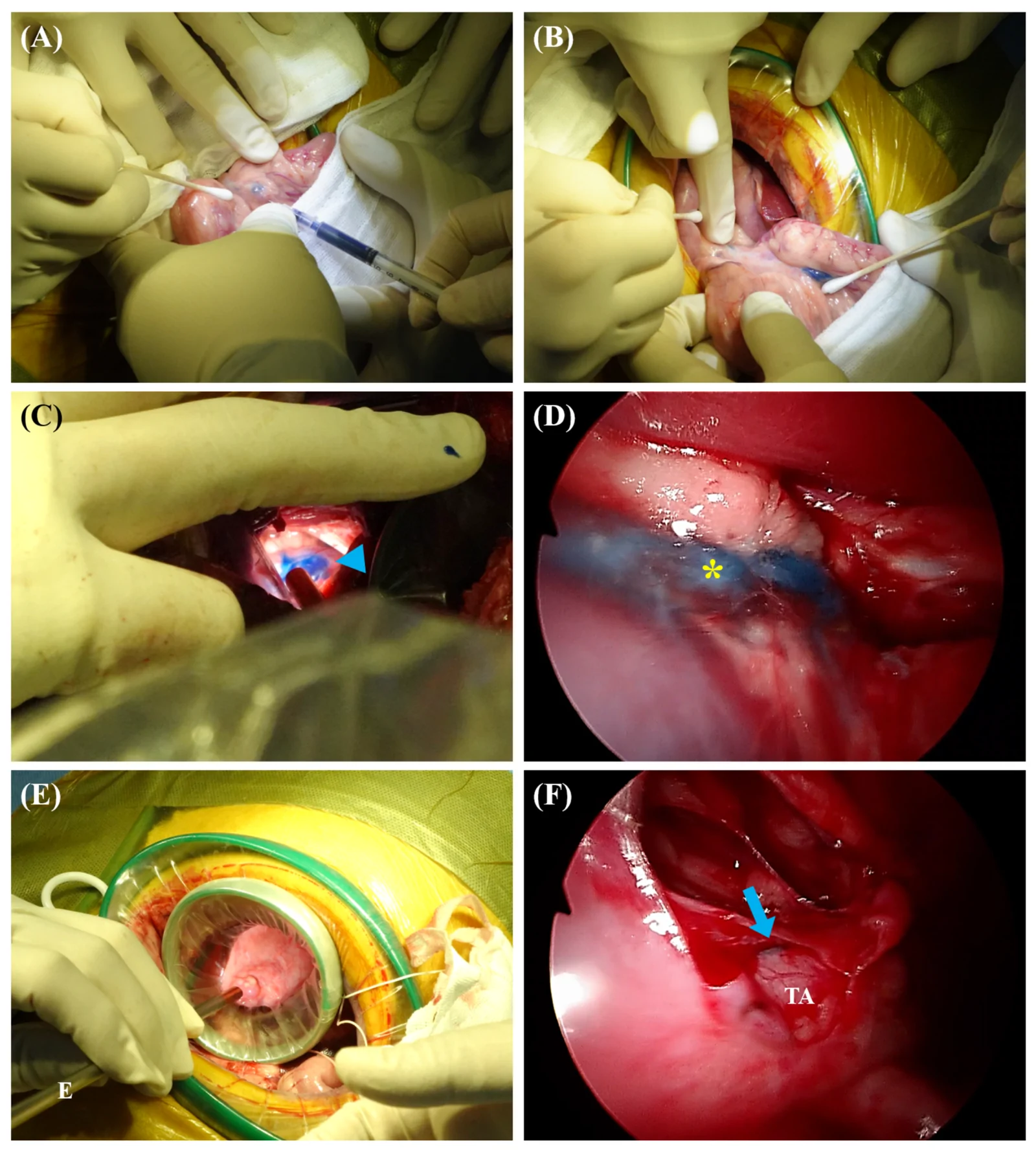
Establishment of endoscope-assisted bicavitary access using double OWR. (**A**,**B**) After the insertion of a larger O-ring wound retractor, methylene blue distribution through the lymphatic vasculature was confirmed following mesenteric lymph node injection. (**C**) Intraoperative endoscopic visualization of the cisterna chyli (blue arrowhead). (**D**) Endoscopic view of the cisterna chyli (yellow asterisk). (**E**) Following circumcostal transdiaphragmatic incision and placement of a smaller O-ring wound retractor to establish thoracic access, the thoracic duct was visualized endoscopically. (**F**) Endoscopic view of the thoracic duct (blue arrow). E, Endoscope; TA, Thoracic aorta.

The dog was positioned in left lateral recumbency, and a right paracostal approach was performed. A right paracostal skin incision was made approximately 2 cm caudal to the last rib and extended dorsoventrally to provide access to the abdominal cavity. Following celiotomy, the abdominal incision was circumferentially retracted using a large OWR (outer diameter 118 mm), which was positioned within the abdominal incision. A mesenteric lymph node was accessed and injected with diluted methylene blue solution (0.5 mL, 1:1 dilution) to facilitate identification of the abdominal lymphatic system and cisterna chyli (Figure 2A–D).

A 3 cm circumcostal incision was made in the peripheral portion of the diaphragm, closely adjacent to the caudal margin of the last rib and sufficiently distant from the aortic hiatus, thereby avoiding direct manipulation of the hiatus and major diaphragmatic structures. After opening the thoracic cavity, controlled ventilation was provided using pressure-controlled ventilation without positive end-expiratory pressure. Peak inspiratory pressure was maintained between 10 and 13 cmH_2_O, and RR was adjusted between 12 and 20 breaths/min to maintain EtCO_2_ between 32 and 48 mmHg. During anesthesia, SpO_2_ remained at ≥95%, MAP was maintained between 65 and 75 mmHg, and FE′Sevo ranged from 1.4% to 2.5%.

**Figure 3:**
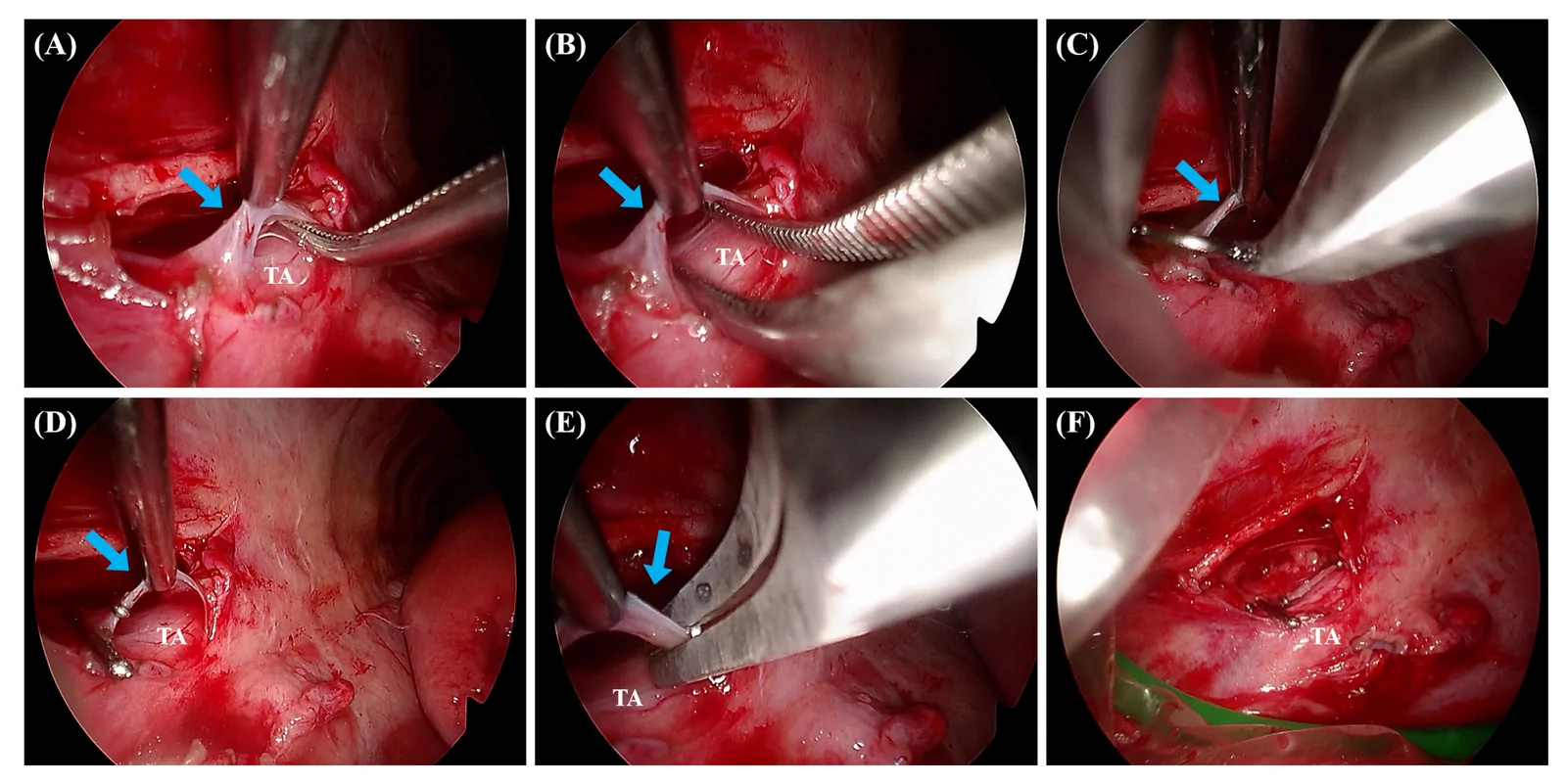
Endoscope-assisted thoracic duct ligation. (**A**,**B**) The thoracic duct (blue arrow) was gently grasped and elevated using DeBakey forceps and dissected free from the thoracic aorta. (**C**–**E**) The thoracic duct (blue arrow) was ligated using three hemostatic clips. (**F**) Completion of thoracic duct ligation. TA, Thoracic aorta.

The smaller OWR (outer diameter 90 mm) was positioned within the diaphragmatic incision to provide circumferential retraction and maintain thoracic access. A rigid endoscope (5.4 mm rigid telescope with 30° optics; Olympus, Tokyo, Japan) was introduced directly through the smaller OWR to visualize the thoracic duct, without an additional trocar or dedicated endoscopic port (Figure 2E,F). No pneumoperitoneum or intentional capnothorax was created, and no gas insufflation was used to expand the pleural space during the procedure. The methylene blue-stained thoracic duct was identified at the level of T10–T12, where it coursed along the right dorsolateral aspect of the aorta. The main thoracic duct was dissected from the surrounding tissues and ligated en bloc with three hemostatic clips (Weck^®^ Horizon™ Titanium Ligating Clips, Medium, Teleflex, Morrisville, NC, USA) at a site caudal to the region of suspected lymphatic leakage identified on CT lymphangiography. The cranial branches were not individually dissected or ligated, as the main thoracic duct was ligated caudal to the suspected leakage region with the intention of interrupting lymphatic flow before it reached this area (Figure 3).

**Figure 4:**
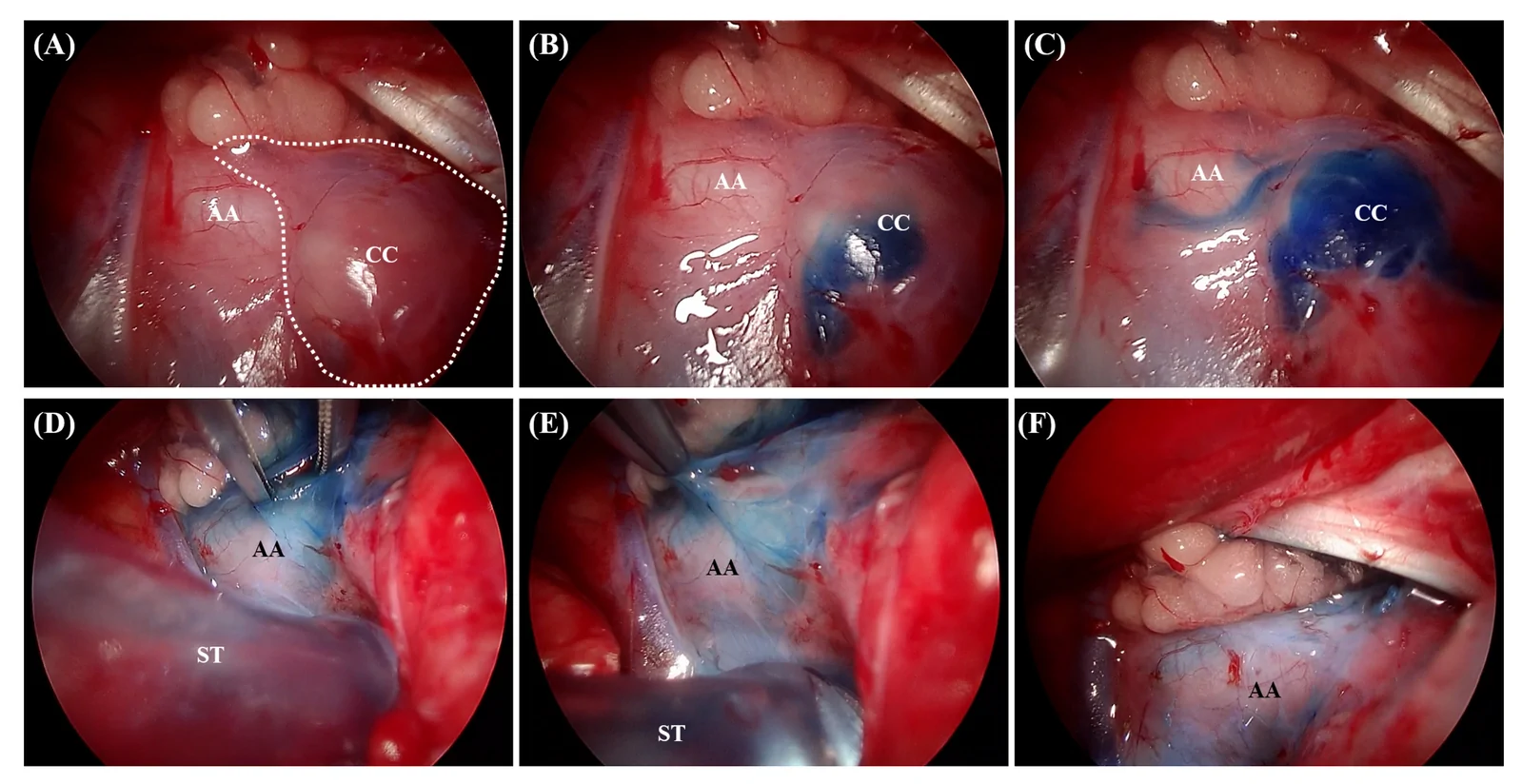
Endoscope-assisted cisterna chyli ablation. (**A**) Endoscopic view of the cisterna chyli (delineated by the dotted line). (**B**,**C**) Additional methylene blue solution was administered for confirmation of lymphatic filling and delineation of the cisterna chyli, with clear endoscopic visualization of dye diffusion. (**D**,**E**) The cisterna chyli was subsequently ablated using DeBakey forceps by carefully grasping and gently dissecting the cisterna chyli wall. (**F**) Completion of cisterna chyli ablation. AA, Abdominal aorta; CC, Cisterna chyli; ST, Suction tip.

**Figure 5:**
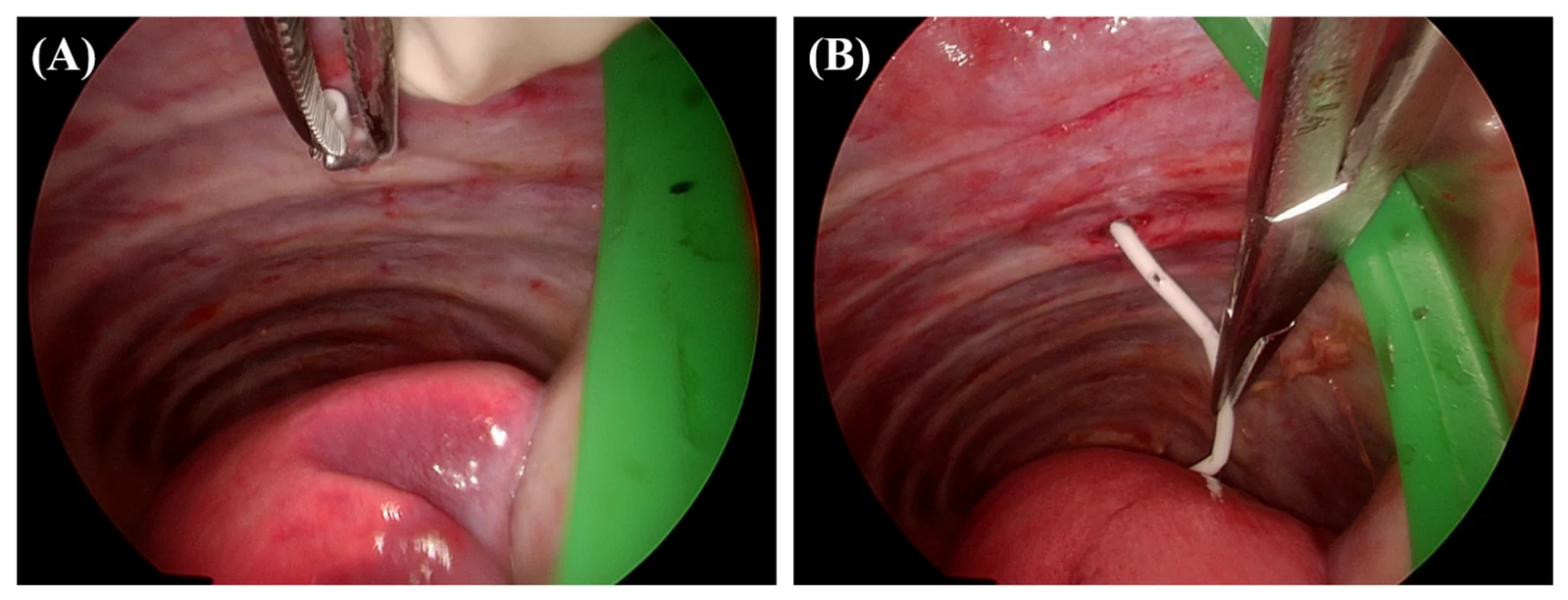
(**A**,**B**) Endoscope-assisted placement of a pleural port.

Additional methylene blue solution (1 mL, diluted 1:3) was administered to confirm lymphatic filling and facilitate visualization of the cisterna chyli (Figure 4A–C). The identified cisterna chyli was carefully grasped and disrupted with DeBakey forceps along its visibly identified extent. In this case, CCA was considered complete when the cisterna chyli could no longer be identified as a distinct lymphatic structure under endoscopic visualization (Figure 4D–F). Endoscopic reassessment of the thoracic cavity was performed to evaluate for residual lymphatic branches and chyle leakage. Following TDL and CCA, endoscopic reassessment revealed no grossly visible dye passage cranial to the ligation site and no residual methylene blue-stained lymphatic branches requiring additional ligation. A pleural port (Canine PleuralPort Kit, Norfolk Vet Products, Skokie, IL, USA) was placed under endoscopic guidance at the completion of TDL and CCA to facilitate the removal of residual pleural effusion and to provide negative-pressure drainage during the early postoperative period (Figure 5). Following completion of the procedure, the diaphragmatic incision was closed with 3-0 polydioxanone (PDS; Ethicon, Cincinnati, OH, USA) in a simple continuous pattern. Before tying the final knot, A 4 Fr red rubber catheter was temporarily inserted through the diaphragmatic incision into the thoracic cavity to evacuate residual air and re-establish negative intrathoracic pressure. The catheter was removed after adequate lung re-expansion and restoration of negative pressure were confirmed, and the diaphragmatic closure was completed. The abdominal wall and skin were then closed routinely. At the completion of surgery, atipamezole (Antisedan, 5 mg/mL; 0.019 mg/kg IM) was administered intraoperatively for reversal of medetomidine-induced sedation.

**Figure 6:**
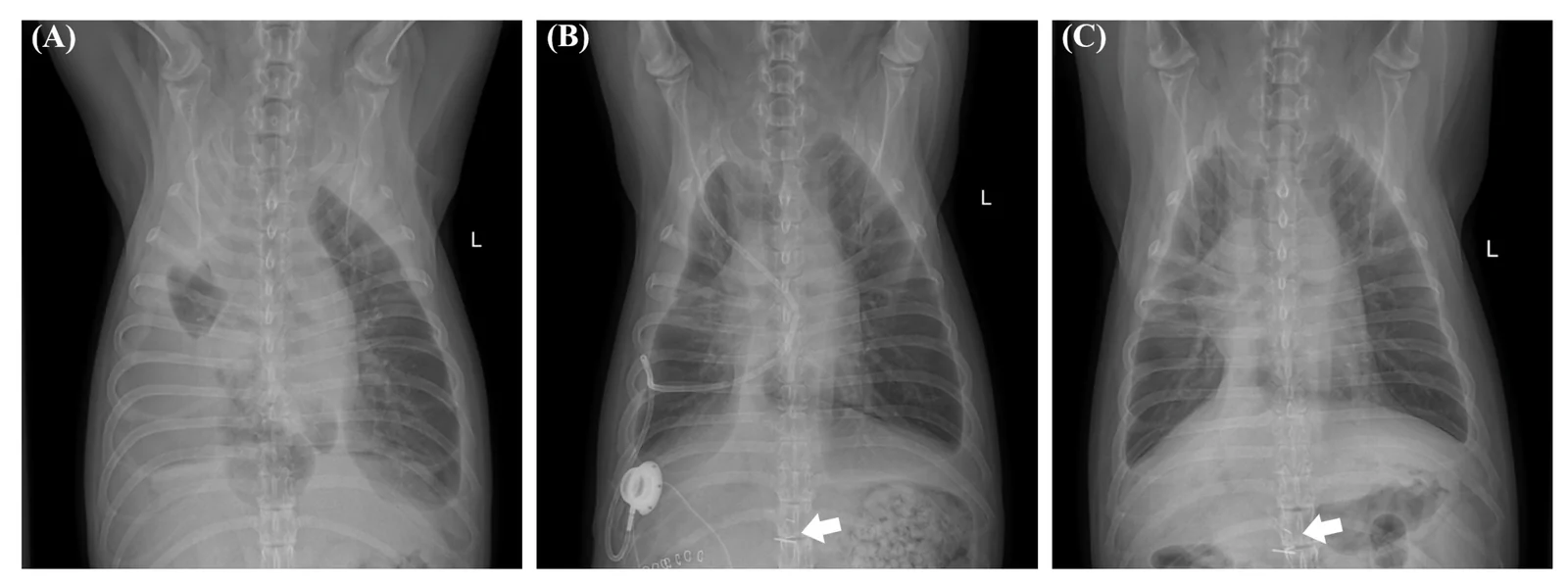
Preoperative and postoperative thoracic radiographic changes. (**A**) Preoperative thoracic radiograph. During the 24 h period immediately preceding surgery, the pleural drainage volume was 13.9 mL/kg/day, measured by thoracocentesis. (**B**) Postoperative day (POD) 20 thoracic radiograph showing reduced pleural effusion, with a drainage volume of 1.75 mL/kg/day obtained via the indwelling pleural port. (**C**) POD 34 thoracic radiograph demonstrating no radiographically detectable pleural effusion. No pleural fluid was obtained during the scheduled 24 h drainage period via the indwelling pleural port (0 mL/kg/day). Arrows indicate the hemostatic clips placed at the ligation site.

Postoperative analgesia consisted of fentanyl CRI (1–3 µg/kg/h, IV) for 2 days, with the dose adjusted according to the patient’s clinical pain assessment, followed by meloxicam (0.1 mg/kg, SC, q24h) for 4 days. Prednisolone was administered orally from postoperative day 7 at 0.5 mg/kg q12h and gradually tapered to 0.5 mg/kg q24h on day 10, 0.3 mg/kg q24h on day 13, and 0.3 mg/kg every other day on day 20, before discontinuation on day 27. Pleural drainage volume was measured daily throughout the postoperative period using the indwelling pleural port. During the 24 h period immediately before surgery, the pleural drainage volume was 13.9 mL/kg/day, measured by thoracocentesis. Following surgery, daily drainage volumes decreased over time, with representative measurements of 12.33 mL/kg/day on postoperative day (POD) 7 and 1.75 mL/kg/day on POD 20, both obtained via the indwelling pleural port. Only time points representing notable changes in drainage volume are reported. By POD 34, no pleural fluid was obtained during the scheduled 24 h drainage period via the indwelling pleural port (0 mL/kg/day), and thoracic radiography showed no radiographically detectable pleural effusion or focal pleural fluid accumulation (Figure 6). These findings were interpreted as resolution of clinically detectable pleural effusion. However, the presence of a small residual or loculated volume of pleural fluid could not be completely excluded. Following pleural port removal on POD 48, approximately 2 mL of visually clear pleural fluid was aspirated through a syringe connected directly to the port during port removal. Biochemical analysis of this residual pleural fluid showed low concentrations of total protein (1.1 g/dL), triglycerides (10 mg/dL), and total cholesterol (33 mg/dL), which were not supportive of chylous fluid. At the final in-person follow-up on POD 48, the dog was clinically stable without signs suggestive of recurrent chylothorax, and thoracic radiography showed no evidence of recurrent pleural effusion. At a subsequent telephone follow-up with the owner on POD 150, the dog was reported to remain clinically stable, with no respiratory abnormalities or other clinical signs suggestive of recurrent chylothorax.

## 3. Discussion

Chylothorax may arise from various underlying conditions, including trauma, neoplasia, cardiovascular disease, and congenital or acquired abnormalities of the lymphatic system [1,3]. In the present case, cardiovascular disease was considered unlikely based on echocardiographic findings, including a normal left atrial size (LA/Ao ratio, approximately 1.2), only mild mitral regurgitation without evidence of myxomatous mitral valve degeneration or prolapse, and no remarkable abnormalities in right atrial or pulmonary arterial blood flow. CT examination also revealed no evidence of a mediastinal mass or other neoplastic lesions. CT lymphangiography demonstrated an abnormal branching pattern at the terminal portion of the thoracic duct, multiple tortuous lymphatic branches, and focal contrast accumulation in the ventral aspect of the right hemithorax, suggesting localized lymphatic leakage near the terminal thoracic duct. Although the underlying cause of these lymphatic abnormalities could not be definitively established, the absence of identifiable traumatic, neoplastic, or cardiovascular causes, together with the abnormal lymphatic anatomy and suspected lymphatic leakage, led us to consider idiopathic chylothorax as the presumptive final diagnosis in this patient.

Endoscope-assisted TDL combined with CCA can provide an alternative means of accessing the thoracic duct and cisterna chyli, but the procedures remain technically demanding and require advanced surgical skills [9]. Potential limitations include difficulty in identifying lymphatic structures and the risk of incomplete TDL or CCA, which may result in persistent collateral lymphatic drainage and recurrence of chylothorax [9]. Procedure-related complications may include anesthetic complications, hemorrhage, pulmonary injury with pneumothorax, poor visualization, equipment failure, and inability to complete the procedure [9,12].

In particular, CCA performed using endoscopic techniques via either a transdiaphragmatic or abdominal approach is associated with distinct technical limitations and potential risks [9]. The transdiaphragmatic approach carries risks of diaphragmatic injury, herniation, and tension pneumothorax associated with gas leakage, and it may be highly operator-dependent [9]. The abdominal approach may be limited by poor visualization of the cisterna chyli, reduced instrument triangulation, and technical difficulty associated with portal placement [9].

The paracostal and transdiaphragmatic approach used in this case is not novel. Previous studies have described combined TDL and CCA through a paracostal transdiaphragmatic approach in experimental and clinical dogs [7], and double OWR have subsequently been applied to this approach [8]. The present case therefore does not introduce a new paracostal or double OWR approach. Rather, the incremental modification was the direct introduction of a rigid endoscope through the smaller OWR to provide intraoperative visualization and reassessment of the thoracic duct and thoracic cavity. This configuration allowed the surgeon to use conventional instruments through the OWR-assisted opening while obtaining endoscopic visualization without a separate trocar or dedicated endoscopic port. However, because this modification was evaluated in only one dog and no objective or comparative measurements of visualization, operative time, surgical exposure, postoperative pain, or procedural performance were performed, its potential advantages cannot be established. Accordingly, the present case should not be interpreted as demonstrating incremental clinical benefit over the previously described double OWR approach.

Although avoidance of an intercostal thoracotomy may reduce the extent of thoracic wall disruption, this theoretical advantage was not objectively assessed in the present case. Intercostal thoracotomy is associated with thoracic wall trauma and potential intercostal nerve injury, which may contribute to postoperative pain [13,14,15,16,17]. Although avoidance of thoracotomy may theoretically reduce thoracic wall trauma, postoperative pain was not objectively assessed in this case, and therefore a reduction in postoperative pain or invasiveness cannot be concluded. Similarly, the absence of major anesthetic or surgical complications cannot be attributed specifically to the OWR-assisted approach. The approach also allowed access to both the abdominal and thoracic cavities during the procedure, facilitating reassessment of the thoracic duct after ligation and placement of the pleural port under endoscopic guidance. These technical features may be useful in selected cases, although their clinical benefits require further comparative evaluation.

Recent studies have investigated TDL combined with CCA and partial pericardiectomy as a more aggressive multimodal surgical approach for idiopathic chylothorax [5,11,18]. However, the indication for partial pericardiectomy remains controversial and case-dependent [19,20]. Therefore, careful patient selection remains important when considering adjunctive partial pericardiectomy. In the present case, TDL and CCA were selected without adjunctive partial pericardiectomy based on the overall clinical, echocardiographic, and CT findings and the absence of clear evidence supporting clinically relevant pericardial disease. However, increased central venous or lymphatic pressure could not be definitively excluded based on these findings alone. Therefore, the decision not to perform partial pericardiectomy was considered a case-specific clinical decision rather than evidence that pericardiectomy was unnecessary in dogs with idiopathic chylothorax.

This report is limited by its single-case design, lack of objective comparative assessment, and absence of postoperative imaging to confirm long-term lymphatic resolution. Although additional owner-reported follow-up was obtained at POD 150, objective long-term recurrence could not be assessed. In addition, no objective measurements of surgical exposure, operative difficulty, postoperative pain, invasiveness, or complication rates were performed. The completeness of lymphatic occlusion following TDL and CCA was assessed only by intraoperative endoscopic visualization and methylene blue findings. In particular, the endpoint for CCA in this case was defined operationally as the point at which the cisterna chyli could no longer be identified as a distinct lymphatic structure under endoscopic visualization. This was a subjective, visually based operative endpoint rather than an objective criterion of complete lymphatic ablation. Postoperative CT lymphangiography was not performed. Therefore, complete interruption of all accessory lymphatic pathways could not be objectively confirmed. Therefore, complete interruption of all accessory lymphatic pathways could not be objectively confirmed. The successful outcome may also have been influenced by surgeon experience, patient selection, and perioperative management, and the individual contributions of the OWR and endoscopic assistance cannot be determined from this case. Nevertheless, endoscope-assisted TDL combined with CCA using double OWR was technically feasible in this dog through a hybrid open transdiaphragmatic approach. Pleural effusion became radiographically undetectable by POD 34 and remained absent on thoracic radiography at POD 48, without major reported perioperative complications. Further comparative studies are required to determine whether endoscopic assistance provides measurable advantages over previously described OWR-assisted techniques.

## 4. Conclusions

Endoscope-assisted TDL combined with CCA using double OWR was technically feasible in this dog with presumed idiopathic chylothorax through a hybrid open transdiaphragmatic approach. This technique permitted endoscopic visualization of the thoracic duct and cisterna chyli while allowing conventional surgical instrumentation through OWR-assisted access. The findings support the use of endoscopic assistance as a potential technical adjunct to previously described OWR-assisted techniques, but further comparative studies are needed to determine whether it provides measurable clinical or procedural advantages.

## Data Availability

The original contributions presented in this study are included in the article. Further inquiries can be directed to the corresponding author.

## Abbreviations

The following abbreviations are used in this manuscript:

TDL	Thoracic duct ligation
CCA	Cisterna chyli ablation
OWR	O-ring wound retractors
CT	Computed tomography
CRI	Constant rate infusion
EtCO_2_	End-tidal carbon dioxide
RR	Respiratory rate
FE′Sevo	Expired fraction of sevoflurane
